# Real-World Data and Evidence in Lung Cancer: A Review of Recent Developments

**DOI:** 10.3390/cancers16071414

**Published:** 2024-04-04

**Authors:** Eleni Kokkotou, Maximilian Anagnostakis, Georgios Evangelou, Nikolaos K. Syrigos, Ioannis Gkiozos

**Affiliations:** Oncology Unit, Third Department of Medicine, “Sotiria” General Hospital for Chest Diseases, National and Kapodistrian University of Athens, 11527 Athens, Greece; maximilian2002@gmail.com (M.A.); grgevangelou@gmail.com (G.E.); nksyrigos@gmail.com (N.K.S.); yiannisgk@hotmail.com (I.G.)

**Keywords:** oncology, real-world data, real-world evidence, epidemiology, safety, efficacy, artificial intelligence, machine learning, data quality, lung cancer

## Abstract

**Simple Summary:**

The use of real-world data (RWD) to generate real-world evidence (RWE) is increasing in oncology. RWD studies provide valuable information to regulators, sponsors, and clinicians. RWD studies rely on collecting and analyzing observational data, offering insights into the practical application of cancer treatment in real-world settings. However, the quality of RWD can compromise the reliability of the RWE. Hybrid methodological analyses that combine the strengths of RCTs and RWD studies, known as R2WE, are being conducted to address these challenges. RWD sources include patient registries and electronic health records (EHRs). High-quality data are essential for generating credible RWE. To obtain RWD, it is necessary to obtain data from relevant sources, clean and harmonize the data, and ensure compliance with the laws and regulatory requirements.

**Abstract:**

Conventional cancer clinical trials can be time-consuming and expensive, often yielding results with limited applicability to real-world scenarios and presenting challenges for patient participation. Real-world data (RWD) studies offer a promising solution to address evidence gaps and provide essential information about the effects of cancer treatments in real-world settings. The distinction between RWD and data derived from randomized clinical trials lies in the method of data collection, as RWD by definition are obtained at the point of care. Experimental designs resembling those used in traditional clinical trials can be utilized to generate RWD, thus offering multiple benefits including increased efficiency and a more equitable balance between internal and external validity. Real-world data can be utilized in the field of pharmacovigilance to facilitate the understanding of disease progression and to formulate external control groups. By utilizing prospectively collected RWD, it is feasible to conduct pragmatic clinical trials (PCTs) that can provide evidence to support randomized study designs and extend clinical research to the patient’s point of care. To ensure the quality of real-world studies, it is crucial to implement auditable data abstraction methods and develop new incentives to capture clinically relevant data electronically at the point of care. The treatment landscape is constantly evolving, with the integration of front-line immune checkpoint inhibitors (ICIs), either alone or in combination with chemotherapy, affecting subsequent treatment lines. Real-world effectiveness and safety in underrepresented populations, such as the elderly and patients with poor performance status (PS), hepatitis, or human immunodeficiency virus, are still largely unexplored. Similarly, the cost-effectiveness and sustainability of these innovative agents are important considerations in the real world.

## 1. Introduction

The utilization of real-world data (RWD) to generate real-world evidence (RWE) in conjunction with interventional clinical trial-based research is rapidly increasing. This burgeoning field, particularly in the context of oncology, has seen a significant number of publications and increased use of RWD in medical regulation in recent years. It is essential to improve the quality of RWE for the benefit of patients, the scientific community, and healthcare authorities.

Oncology research presents a multitude of particularities, including specific variables, biomarkers, therapies, and outcomes, which are not adequately addressed by the existing reporting guidelines. Furthermore, contemporary technologies such as artificial intelligence (AI), machine learning (ML), and deep learning (DL) have been integrated into various stages of data analysis in RWE studies. Although guidelines for interventional studies involving AI are now available [1,2], similar guidance specifically tailored for RWE research remains absent.

RWD studies have several advantages, such as increased sample sizes, quicker achievement of research objectives, and reduced costs, compared to conventional clinical trials [3,4,5]. Nevertheless, when conducting RWD studies, several challenges must be addressed, such as the accessibility and protection of data, compliance with relevant laws and regulations, and the necessity for meticulous study design and analysis.

## 2. Role of RWD in Oncology

**Figure 1:**
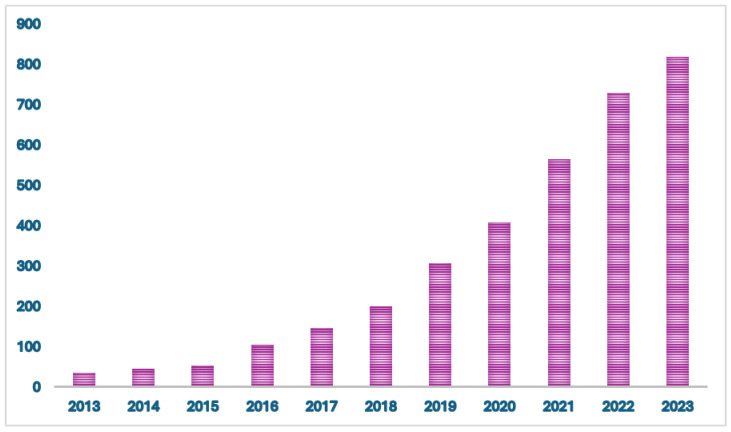
Number of citations/year of real-world evidence in oncology between January 2013 and January 2024.

Real-world data (RWD) studies are increasingly being utilized as alternative sources of evidence in clinical cancer research (Figure 1). These studies provide valuable information to inform the decisions of regulators, sponsors, and clinicians. RWD studies primarily focus on collecting and analyzing observational data, which offers insights into the practical application of cancer treatment in real-world settings.

Low-quality RWD sometimes compromise the reliability of real-world evidence (RWE). In such cases, randomized controlled trials (RCTs) are necessary to answer specific research questions definitively. Hybrid methodological analyses that combine the strengths of RCTs and RWD studies, known as R2WE, are currently being conducted to address these challenges. The European Organization for Research and Treatment of Cancer (EORTC) is developing a strategy to build solid, high-quality RWE by prioritizing realistic clinical trials. This approach aims to provide evidence to support new therapeutic approaches in clinical practice [6].

Research using RWD has several advantages, including access to larger datasets, greater generalizability, and shorter study duration. However, obtaining data and ensuring compliance with laws and regulatory requirements can be challenging. Despite these obstacles, RWD studies have the potential to address evidence gaps and provide crucial information about the effects of cancer treatment in real-world settings [6,7].

RWD sources include patient registries and electronic health records (EHRs). Patient registries are structured systems that gather specific information about patients with a particular condition or treatment, whereas EHRs are digital versions of patients’ medical records. Other sources of RWD include patient questionnaires, mobile devices, smartphones, and social media [8,9].

High-quality data are the foundation for credible RWE. To generate RWE, it is essential to obtain data from relevant RWD sources, clean and harmonize the data, and link them to fill in gaps. Additionally, the data must include endpoints relevant to the research question. Quality criteria must be applied throughout the process of generating RWE, from data sources to processing, to ensure that appropriate use cases are defined.

The importance of patient follow-up during daily clinical practice in the field of oncology is becoming increasingly recognized as a valuable means of data collection. Such follow-up studies offer valuable insights into the safety and efficacy of interventions in specific patient populations, including those with chronic viral diseases, brain metastases, or poor performance status. By incorporating data analysis and evaluating the impact on healthcare budgets, real-world population follow-up studies have the potential to inform more appropriate treatment choices and may even be considered part of the regulatory approval process in the future [10].

## 3. Potential Use of RWE

Regulatory-grade real-world evidence (RWE) has the potential to provide critical information for informed decision making by clinicians, patients, and regulatory authorities. Traditional Phase IV and other post-marketing studies can be burdensome and face numerous obstacles in patient enrollment, such as evolving practice patterns. Well-designed RWE studies can generate innovative hypotheses for future research in basic sciences, drug development, health outcomes, and clinical trials. Longitudinal RWE could potentially aid the identification of rare side effects across extensive populations. By employing thorough RWE studies, it may be possible to uncover adverse event trends in real time rather than relying solely on voluntary reporting. Through a comprehensive assessment of both structured and unstructured real-world data (RWD) for individual patients, RWE can rigorously document safety and effectiveness at the necessary level of quality and detail to support label expansion.

When a new oncological therapy is in the process of development, it is subject to various decision points that determine whether it will continue to be developed. The use of RWE can aid in optimizing these decisions during predevelopment and in guiding clinical development strategies by clarifying unmet needs in the real world. The incorporation of RWE into clinical development can also play a role in the planning and execution of clinical trials. By offering insights into specific populations, RWE can aid in reducing the excessively restrictive exclusion criteria. Furthermore, knowledge of the prevalence patterns of potential trial candidates, such as rare cancers that progress despite chemotherapy, can facilitate patient recruitment for clinical trials. The use of synthetic control arms based on RWE is also being investigated, particularly for cancers with well-established standards of care, poor prognoses, and low incidences (e.g., small-cell lung cancer). In contrast to historical controls, synthetic controls may be more recent, which can help to account for changes in supportive care over time.

Treatment decisions are often determined by the risk–benefit ratio for each individual patient. Although it is impossible to completely eliminate clinical uncertainty, the utilization of RWE can aid in refining this assessment and facilitating personalized medicine tailored to both the patient and tumor. The extent and significance of potential RWE use cases necessitate stringent quality assessments, particularly when utilized for regulatory decision making.

The complexities of lung cancer treatment, coupled with the diverse range of therapeutic options currently available, require delicate and informed clinical decision making on the part of healthcare providers and those responsible for resource allocation (e.g., payers and regulators) [11]. This highlights the need for rapid and ongoing insights into these decisions. Such insights are typically provided by data from randomized controlled trials (RCTs) and RWE. RWE can offer information that would not be readily obtainable through RCTs, generating data that reflect routine clinical practice in larger, real-life treatment populations. In the field of lung cancer drug development, there are many examples of the usefulness of RWE in providing complementary data to reinforce and support clinical trial data. For instance, recent real-world studies have evaluated the safety and/or effectiveness of lung cancer treatments, often in patient cohorts ineligible for clinical trials [12,13,14,15]; investigated the real-world burden of lung cancers and related treatment patterns and survivorship [13,14,16,17,18]; and assessed treatment-related costs/healthcare resource utilization (HCRU) [13,14,17,19]. Therefore, data from both observational real-world studies and traditional RCTs are important in informing the best clinical practice, and regulatory bodies recognize that these two methodologies are complementary in both the pre- and post-authorization stages of drug development [14].

## 4. RWE in Cancer Drug Development

The current regulatory framework of the FDA affords sufficient flexibility to integrate novel forms of clinical evidence into decision-making processes [20]. To this end, efforts should concentrate on devising suitable study designs and strategies for acquiring high-quality data from EHRs in the context of real-world data utilization.

(1) Real-world evidence for digital pharmacovigilance

Regulatory agencies primarily rely on passive surveillance to ensure post-market pharmacovigilance. This approach entails the analysis of voluntary reports of adverse events submitted by healthcare professionals and patients as well as mandatory reporting from pharmaceutical companies [21]. However, passive reporting has several limitations, including the influence of extraneous factors, such as media attention and the length of time a product has been on the market. To address these challenges, the FDA established the Sentinel Initiative, which seeks to develop an active surveillance system that proactively investigates real-world data (RWD) to detect new safety signals [22,23]. The advent of advanced information technology presents an opportunity to create an integrated approach that leverages RWD from electronic health records (EHRs) and patient-generated sources, such as mobile applications and Internet search logs, to modernize pharmacovigilance [24,25]. By adopting a digital pharmacovigilance system that merges RWD from healthcare providers through electronic health records (EHRs) and online platforms that are used by patients, researchers and experts in the pharmaceutical industry and regulatory authorities can develop a proactive monitoring system, permitting the application of protective measures to recognize certain safety concerns. In a digital drug safety monitoring system, the efficiency of safety indicators can be analyzed using techniques such as data mining, like proportional reporting ratios and empirical Bayesian geometric mean scores, which have already been utilized by regulatory authorities like the FDA [26,27]. Furthermore, a digital system that integrates various streams of real-world data can use deep learning methods with the help of artificial intelligence and natural language processing to improve safety signal detection methods [28,29,30].

(2) Utilizing RWE to investigate disease progression and establish external control

Disease history encompasses the course from a symptom-free phase to the appearance of various symptoms and continues to the phase where either the disease is cured, or the patient is deceased [31]. For better understanding the course of the disease, two types of variables must be examined that influence the probability of the disease to progress from the asymptomatic to symptomatic phase [32]. RWD present a valuable opportunity to investigate the covariates that affect the natural history of diseases in populations where a significant portion is regularly monitored and treated. For instance, retrospective analysis of EHR data can be employed to identify covariates that contribute to the onset of cancer in healthy individuals. This analysis can help elucidate the patient and environmental factors that influence disease occurrence. Similarly, examining EHR data retrospectively can aid in identifying covariates associated with cancer progression from the asymptomatic to the symptomatic stage. Such analyses offer valuable knowledge on the structure of prospective clinical trials in the future that will evaluate the effect of screening for cancer and early treatment of patients.

Utilizing external control data in specific clinical trials, such as single-arm trials, may improve the development of relative standards for regulatory decision making, especially in the context of severe diseases characterized by substantial medical requirements that remain unresolved such as advanced malignancies [33]. If preliminary clinical evidence from a single-arm trial indicates a significant treatment effect, evaluating outcomes in comparable patient groups using RWD can offer a reliable assessment of the safety and efficacy of available therapies for comparison.

Advancements in genomic sequencing and computational proteomics have led to the identification of a growing number of rare tumor variants resulting from somatic mutations, proteomic signatures, and alterations in cell signaling pathways with oncogenic potential [34,35,36]. Retrospective analysis of RWD can provide an adequate approach to the evaluation of biomarkers and their prognostic significance regarding disease outcomes in rare subgroups during clinical development. Clinical results from RWD registries can be connected to genomic and proteomic profiles for predicting outcomes and establishing guidelines. This will require enhancing big data analytics capabilities to effectively analyze complex and rare patterns identified by multi-omic pipelines, aiming to improve patient care and outcomes.

(3) Observational real-world studies

There is a growing convergence between the outcomes of randomized controlled trials (RCTs) and well-designed observational studies, which presents an opportunity to develop robust methodologies that support EHR-based observational research [20,37,38]. Data collection from real-world settings through observational studies can contribute valuable information, which can be utilized in randomized controlled trials or regulatory decision making. Furthermore, observational studies offer an opportunity to evaluate the effectiveness and safety of treatments in patient populations that are often excluded from conventional cancer clinical trials. New regulatory incentives for drug developers to submit RWD of patients excluded from conventional clinical trials can improve the generalizability of FDA label information, enabling prescribers to make informed treatment decisions [39].

(4) Practical clinical studies

EHRs serve as primary instruments for conducting practical clinical trials (PCTs). EHRs are widely accessible tools in the healthcare sector that can aid in establishing a clinical trial program based within the point of care and linked to patients digitally through innovative technologies like sensors and mobile apps. Through facilitating the deliberate gathering of pertinent clinical data that accurately represent the diverse range of cancer patients, EHRs play a role in bringing real-world evidence to pharmaceutical research, while emphasizing advancements in quality, patient safety, and value in cancer treatment delivery [40]. 

In the realm of cancer drug development, PCTs present numerous advantages compared to traditional clinical trials, which are typically limited to specialized facilities with the required resources and capacity to maintain research initiatives. The limited involvement rates in cancer trials, particularly among minority groups, the elderly, individuals with low income, and those living in rural areas (less than 5%), underscore the obstacles presented by the division of clinical research across geographically dispersed locations [20,41,42,43]. The primary reason for the low participation in cancer trials is the obstacles to accessing convenient experimental treatments, rather than patient preferences [44,45]. PCTs are capable of complying with standard principles of scientific, ethical, legal, and regulatory oversight in the field of clinical studies, while simultaneously extending the availability of experimental therapies in a manner that is both secure and efficient.

(5) Evaluating risks to the internal validity of real-world studies

The internal validity of studies conducted in real-world settings, particularly those utilizing nonrandomized designs, necessitates the effective management of bias stemming from provider–patient interactions, methodologies employed in data collection and processing, and the diverse practice patterns present within regional healthcare systems.

## 5. RWD on ICI Outcomes and Safety

In recent years, the introduction of immune checkpoint inhibitors (ICIs) has significantly improved the treatment of lung cancer. Anti-PD1 (pembrolizumab and nivolumab) and anti-PD-L1 (atezolizumab and durvalumab) agents are capable of overcoming the immune evasion mechanisms used by tumors and restoring the immune system’s antitumor response. At the beginning, ICIs were used as primary or secondary therapies for patients with advanced-stage disease, both for those selected based on PD-L1 expression (such as pembrolizumab) and for the overall patient population. Afterward, durvalumab was integrated as a consolidation therapy in a treatment algorithm for PD-L1-positive locally advanced non-small-cell lung cancer (NSCLC). Currently, ICIs are employed as adjuvant and neoadjuvant treatments.

**Figure 2:**
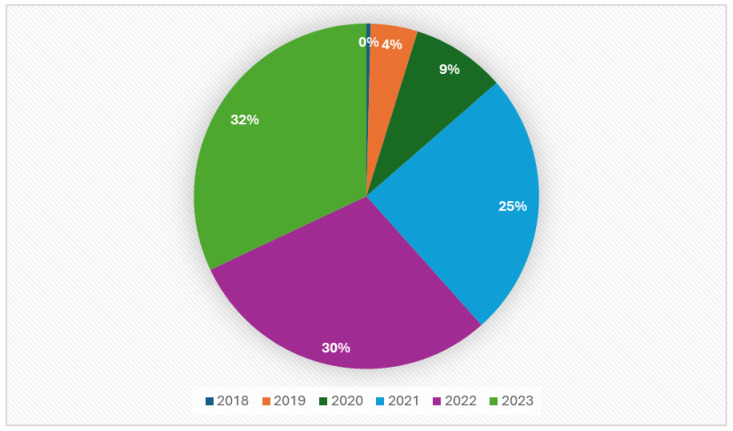
Amount of real-world data on ICIs between 2018 and 2024.

Randomized controlled trials (RCTs) are widely regarded as the most robust form of evidence and, therefore, serve as the gold standard for evaluating the efficacy of an intervention. Nonetheless, the translation of this evidence into real-life clinical practice can be problematic, as a substantial number of patients encountered in everyday practice are often underrepresented in RCTs. In light of the use of ICIs as the standard of care for lung cancer, oncologists are confronted with the dearth of data pertaining to patient subsets that are typically excluded from pivotal clinical trials. In particular, it is of utmost importance to gather information concerning the safety and efficacy of ICIs in individuals with chronic viral diseases, as well as in those presenting with brain metastases or an Eastern Cooperative Oncology Group (ECOG) performance status (PS) of 2 or worse (Figure 2).

The majority of data available on the application of immunotherapy in lung cancer pertain to its efficacy, as measured by the objective response rate (ORR), overall survival (OS), and progression-free survival (PFS), as well as its safety profile, with respect to the most frequently utilized ICIs in clinical practice.

## 6. Real-World Evidence on Special Populations

(1) The elderly

The typical age of individuals at the time of lung cancer diagnosis is 70 years old [46]. It is worth noting that the elderly population is often underrepresented in randomized controlled trials (RCTs). Analysis of the studies showed that the effectiveness of ICI monotherapy was not substantially different among elderly and younger patients. Furthermore, age did not have any effect on tolerability [34,47,48,49,50]. Muchnik et al. observed that the frequency of immune-related colitis was increased among those who were over the age of 80 [34].

(2) ECOG performance status 2

The primary pivotal trials for ICIs in lung cancer strictly excluded patients with poor performance status (PS), confining the inclusion criteria to PS 0 or 1 according to the ECOG classification, whereas only a limited number of clinical trials enrolled patients with PS 2 [51,52]. Nevertheless, both the European Medicines Agency (EMA) and the Food and Drug Administration (FDA) granted approval for the four available ICIs without regard to the PS of patients. As a result, the use of ICIs in clinical practice for patients with PS 2 has been permissible, leading to the accumulation of real-world data.

The absence of solid data from RCTs warrants caution regarding the use of ICIs in patients with poor PS. Even if ICIs have a favorable safety profile in case of a lack of survival benefits, it may not be sufficient to justify the significant expenses of their use [53]. The PePS2 trial evaluated the safety and tolerability of pembrolizumab in treating NSCLC patients with an ECOG PS of 2 [54]. The trial has co-primary endpoints that include measuring the durable clinical benefit (DCB), objective response rate (ORR), and incidence of dose interruptions or discontinuations due to immune-related adverse events (irAEs). A preliminary analysis of data from a subgroup of 60 patients revealed a DCB rate of 33%, an ORR of 30%, and an irAE incidence of 8%. Although these initial results are encouraging, when analyzing the results of survival rates it is crucial to proceed with caution, as the median PFS was 5.4 months and OS was 11.7 months, and only 15% of patients (9 out of 60) received first-line pembrolizumab, resulting in no responses and a PFS of 1.9 months. In a retrospective study conducted by Facchinetti et al., the outcomes of patients with PS 2 who received first-line pembrolizumab treatment were evaluated. The results indicated that patients with PS 2 related to comorbidity had a median overall survival (OS) of 11.8 months, while those with PS 2 driven by lung cancer had a median OS of 2.8 months. The hazard ratio (HR) for OS was 0.5 (*p* = 0.001) in favor of the former group [55].

(3) Central nervous system metastases

The central nervous system (CNS) is a common site of metastases in patients with non-small-cell lung cancer (NSCLC), with an estimated incidence of brain metastases (BMs) of approximately 40%. Patients with BM often experience symptoms, necessitating treatment with corticosteroids, and have a poor prognosis. Consequently, this individual population is not adequately represented in clinical trials, and patients with BM are included only when their CNS disease is not active and does not need immediate treatment. In clinical studies evaluating immunotherapy in lung cancer, the percentage of patients with inactive and asymptomatic BMs ranges from 6% to 17.5%, and no preplanned analysis of CNS metastasis subgroups has been conducted [56,57,58,59,60].

Currently, the available empirical data are limited. However, a study conducted by Pasello has provided valuable insights by examining 255 individuals with BM who were participants in a multicenter, prospective research project and were administered ICIs [61]. The study population comprised approximately 40% individuals with active BM and 14% symptomatic patients. Even though patients with CNS metastasis have a reduced probability of disease control and an increased risk of progressive brain disease, a multivariate analysis of a study considering treatment with steroids and patient PS showed that the existence of BM does not independently predict OS [61]. Furthermore, the application of cranial radiotherapy, whether in the use of whole-brain or stereotactic radiotherapy, did not demonstrate a significant impact on survival. Including patients with CNS metastasis, the Italian Expanded Access Program (EAP) used nivolumab and demonstrated no disparities in OS between the squamous and non-squamous populations when compared with the general population OS [62,63,64]. The French EAP with nivolumab, which included 130 patients with BM, yielded similar outcomes [65]. Generally, the body of RWD derived from patients with NSCLC with CNS metastasis who have been treated with immunotherapy offers more compelling evidence of both safety and efficacy than RCTs in this particular patient population.

(4) Patients with pre-existing autoimmune disorders

ICIs act on molecular pathways involved in physiological immune self-tolerance. These treatments are associated with irAEs, which are essentially new autoimmune disorders triggered by therapy. Owing to this risk, patients with pre-existing autoimmune diseases (AIDs) were excluded from clinical trials for ICIs, except those with vitiligo, type I diabetes mellitus, or residual hypothyroidism that only requires hormone replacement. This exclusion was based on fear of unacceptable immune reactions and severe toxicities. Individuals with these disorders are susceptible to malignant tumors, particularly lung cancer [66]. Almost one-fifth of all patients with lung cancer have an underlying AID [67,68]. Several retrospective studies have investigated the potential risks and benefits of immunotherapy in a specific subset of patients. In a noteworthy study, Leonardi et al. examined 56 patients with advanced NSCLC and an AID who were treated with either anti-PD-1 or anti-PD-L1 therapy [69]. The researchers reported that the incidence of irAEs was similar to that observed in clinical trials. Additionally, they noted that AID exacerbations occurred in only a minority of patients, particularly those who were already experiencing symptoms of their AID at the time of initiating immunotherapy. A comprehensive retrospective study was conducted on a substantial cohort of patients (n = 751) diagnosed with advanced solid malignancies who were treated with anti-PD-1 agents. This study aimed to assess the safety and efficacy of these treatments in relation to the presence of pre-existing AIDs [68]. Two-thirds of the patients were diagnosed with NSCLC. This study revealed that the incidence of irAEs of any grade was higher in patients with pre-existing AIDs, regardless of whether the AIDs were symptomatic. However, no significant differences were observed in the incidence of grade 3–4 irAEs, ORR, PFS, or OS between the two groups. Additionally, it was found that around half of the patients who had prior AIDs experienced a recurrence of their autoimmune condition, based on their subtype of AID. The rate of occurrence varied from 10% for rheumatologic disorders to 100% for gastrointestinal and hepatic diseases [68]. According to these real-world data, pre-existing AIDs should not be immediately considered as absolute contraindications for immunotherapy.

(5) Patients with chronic viral diseases

The majority of clinical trials involving ICIs for NSCLC have typically excluded patients with chronic viral infections, including HIV, HBV, and HCV. Concerns regarding potential viral reactivation and the need for antiviral therapy raise questions about treatment efficiency and safety. However, retrospective case series have shown that ICI treatment is safe for patients with NSCLC who are HIV-positive, with no evidence of viral rebound, and with similar safety profiles among 30 patients with advanced NSCLC [51,70]. There is limited information available on the use of ICIs in patients with NSCLC and HBV or HCV. In a retrospective study, 10 patients with NSCLC and HBV or HCV who received immunotherapy had similar toxicity and efficacy rates to those without viral infections [71], without any impact on viral load or replication.

## 7. Efficacy–Effectiveness Gap in Metastatic NSCLC

Several studies investigated the effectiveness of systemic treatment in RWD compared to efficacy data from clinical trials, referred to as the efficacy–effectiveness gap [72,73]. The survival of patients with metastatic NSCLC who are treated with chemotherapy or targeted therapy in real-world practice is almost one-quarter shorter than that of patients who participate in clinical trials. The difference can be partially explained by the patients’ performance status, earlier discontinuation, and fewer subsequent lines of treatment [72]. A comparative analysis was conducted to assess the progression-free survival (PFS) and overall survival (OS) of patients who received first-line (1L) pembrolizumab and second-line (2L) nivolumab, along with the inclusion of clinical trial data [73]. It was additionally shown that the occurrence of obtaining successive lines of treatment was less frequent in real-world settings compared to in clinical trials. There was no evidence of a difference in PFS of patients with stage IV NSCLC that receive immunotherapy among findings from RWD and clinical studies. But a distinction in OS was observed with regard to 1L pembrolizumab, potentially due in part to a reduced number of patients advancing to a subsequent course of treatment in practice [73].

## 8. Conclusions and Future Directions

The use of real-world data (RWD) in oncology is increasing to generate real-world evidence (RWE). RWD studies offer valuable information to regulators, sponsors, and clinicians. These studies rely on observational data and provide insights into real-world cancer treatment. Hybrid methodological analyses such as R2WE are being conducted to address the challenges of RWD quality. The European Organization for the Research and Treatment of Cancer (EORTC) is developing a strategy to create high-quality RWE by prioritizing realistic clinical trials.

## References

[1] Dlamini Z., Francies F.Z., Hull R., Marima R. (2020). Artificial intelligence (AI) and big data in cancer and precision oncology. Comput. Struct. Biotechnol. J..

[2] Adir O., Poley M., Chen G., Froim S., Krinsky N., Shklover J., Shainsky-Roitman J., Lammers T., Schroeder A. (2020). Integrating Artificial Intelligence and Nanotechnology for Precision Cancer Medicine. Adv. Mater..

[3] Pregelj L., Hwang T.J., Hine D.C., Siegel E.B., Barnard R.T., Darrow J.J., Kesselheim A.S. (2018). Precision Medicines Have Faster Approvals Based on Fewer and Smaller Trials Than Other Medicines. Health Aff..

[4] Agrawal S., Arora S., Amiri-Kordestani L., de Claro R.A., Fashoyin-Aje L., Gormley N., Kim T., Lemery S., Mehta G.U., Scott E.C. (2023). Use of Single-Arm Trials for US Food and Drug Administration Drug Approval in Oncology, 2002–2021. JAMA Oncol..

[5] Davis C., Naci H., Gurpinar E., Poplavska E., Pinto A., Aggarwal A. (2017). Availability of evidence of benefits on overall survival and quality of life of cancer drugs approved by European Medicines Agency: Retrospective cohort study of drug approvals 2009-13. BMJ.

[6] Saesen R., Lacombe D., Huys I. (2021). Design, organisation and impact of treatment optimisation studies in breast, lung and colorectal cancer: The experience of the European Organisation for Research and Treatment of Cancer. Eur. J. Cancer.

[7] Istl A.C., Gronchi A. (2022). Neoadjuvant Therapy for Primary Resectable Retroperitoneal Sarcomas—Looking Forward. Cancers.

[8] Makady A., de Boer A., Hillege H., Klungel O., Goettsch W. (2017). What Is Real-World Data? A Review of Definitions Based on Literature and Stakeholder Interviews. Value Health.

[9] Sherman R.E., Anderson S.A., Dal Pan G.J., Gray G.W., Gross T., Hunter N.L., LaVange L., Marinac-Dabic D., Marks P.W., Robb M.A. (2016). Real-World Evidence—What Is It and What Can It Tell Us?. N. Engl. J. Med..

[10] Saesen R., Van Hemelrijck M., Bogaerts J., Booth C.M., Cornelissen J.J., Dekker A., Eisenhauer E.A., Freitas A., Gronchi A., Hernán M.A. (2023). Defining the role of real-world data in cancer clinical research: The position of the European Organisation for Research and Treatment of Cancer. Eur. J. Cancer.

[11] Berger M.L., Sox H., Willke R.J., Brixner D.L., Eichler H., Goettsch W., Madigan D., Makady A., Schneeweiss S., Tarricone R. (2017). Good practices for real-world data studies of treatment and/or comparative effectiveness: Recommendations from the joint ISPOR-ISPE Special Task Force on real-world evidence in health care decision making. Pharmacoepidemiol. Drug Saf..

[12] Kawachi H., Fujimoto D., Morimoto T., Ito M., Teraoka S., Sato Y., Nagata K., Nakagawa A., Otsuka K., Tomii K. (2018). Clinical Characteristics and Prognosis of Patients With Advanced Non–Small-cell Lung Cancer Who Are Ineligible for Clinical Trials. Clin. Lung Cancer.

[13] Arunachalam A., Li H., Bittoni M.A., Camacho R., Cao X., Zhong Y., Lubiniecki G.M., Carbone D.P. (2018). Real-World Treatment Patterns, Overall Survival, and Occurrence and Costs of Adverse Events Associated With Second-Line Therapies for Medicare Patients With Advanced Non–Small-Cell Lung Cancer. Clin. Lung Cancer.

[14] Ekman S., Griesinger F., Baas P., Chao D., Chouaid C., O'Donnell J.C., Penrod J.R., Daumont M., Lacoin L., McKenney A. (2019). I-O Optimise: A novel multinational real-world research platform in thoracic malignancies. Futur. Oncol..

[15] Fukui T., Okuma Y., Nakahara Y., Otani S., Igawa S., Katagiri M., Mitsufuji H., Kubota M., Hiyoshi Y., Ishihara M. (2019). Activity of Nivolumab and Utility of Neutrophil-to-Lymphocyte Ratio as a Predictive Biomarker for Advanced Non–Small-Cell Lung Cancer: A Prospective Observational Study. Clin. Lung Cancer.

[16] Buck P.O., Saverno K.R., Miller P.J., Arondekar B., Walker M.S. (2015). Treatment Patterns and Health Resource Utilization Among Patients Diagnosed With Early Stage Resected Non–Small Cell Lung Cancer at US Community Oncology Practices. Clin. Lung Cancer.

[17] Wallington M., Saxon E.B., Bomb M., Smittenaar R., Wickenden M., McPhail S., Rashbass J., Chao D., Dewar J., Talbot D. (2016). 30-day mortality after systemic anticancer treatment for breast and lung cancer in England: A population-based, observational study. Lancet Oncol..

[18] Nadler E., Espirito J.L., Pavilack M., Boyd M., Vergara-Silva A., Fernandes A. (2018). Treatment Patterns and Clinical Outcomes Among Metastatic Non–Small-Cell Lung Cancer Patients Treated in the Community Practice Setting. Clin. Lung Cancer.

[19] Andreas S., Chouaid C., Danson S., Siakpere O., Benjamin L., Ehness R., Dramard-Goasdoue M.-H., Barth J., Hoffmann H., Potter V. (2018). Economic burden of resected (stage IB-IIIA) non-small cell lung cancer in France, Germany and the United Kingdom: A retrospective observational study (LuCaBIS). Lung Cancer.

[20] Khozin S., Blumenthal G.M., Pazdur R. (2017). Real-world Data for Clinical Evidence Generation in Oncology. JNCI J. Natl. Cancer Inst..

[21] Edwards I.R. (2012). An agenda for UK clinical pharmacology: Pharmacovigilance. Br. J. Clin. Pharmacol..

[22] Food and Drug Administration (2017). Sentinel Initiative. http://www.fda.gov/Safety/FDAsSentinelInitiative/ucm149340.htm.

[23] Food and Drug Administration (2008). Sentinel Initiative: A National Strategy for Monitoring Medical Product Safety. http://www.fda.gov/Safety/FDAsSentinelInitiative/ucm089474.htm.

[24] White R.W., Harpaz R., Shah N.H., DuMouchel W., Horvitz E. (2014). Toward Enhanced Pharmacovigilance Using Patient-Generated Data on the Internet. Clin. Pharmacol. Ther..

[25] Salathé M. (2016). Digital Pharmacovigilance and Disease Surveillance: Combining Traditional and Big-Data Systems for Better Public Health. J. Infect. Dis..

[26] Evans S.J.W., Waller P.C., Davis S. (2001). Use of proportional reporting ratios (PRRs) for signal generation from spontaneous adverse drug reaction reports. Pharmacoepidemiol. Drug Saf..

[27] Dumouchel W. (1999). Bayesian Data Mining in Large Frequency Tables, with an Application to the FDA Spontaneous Reporting System. Am. Stat..

[28] Nikfarjam A., Sarker A., O’connor K., Ginn R., Gonzalez G. (2015). Pharmacovigilance from social media: Mining adverse drug reaction mentions using sequence labeling with word embedding cluster features. J. Am. Med. Inform. Assoc..

[29] Cocos A., Fiks A.G., Masino A.J. (2017). Deep learning for pharmacovigilance: Recurrent neural network architectures for labeling adverse drug reactions in Twitter posts. J. Am. Med. Inform. Assoc..

[30] Graves A., Wayne G., Reynolds M., Harley T., Danihelka I., Grabska-Barwińska A., Colmenarejo S.G., Grefenstette E., Ramalho T., Agapiou J. (2016). Hybrid computing using a neural network with dynamic external memory. Nature.

[31] de la Paz M.P., Villaverde-Hueso A., Alonso V. (2010). Rare diseases epidemiology research. Adv. Exp. Med. Biol..

[32] Brookmeyer R. (1990). Statistical problems in epidemiologic studies of the natural history of disease. Environ. Health Perspect..

[33] Davi R., Mahendraratnam N., Chatterjee A., Dawson C.J., Sherman R. (2020). Informing single-arm clinical trials with external controls. Nat. Rev. Drug Discov..

[34] Leiserson M.D.M., Vandin F., Wu H.-T., Dobson J.R., Eldridge J.V., Thomas J.L., Papoutsaki A., Kim Y., Niu B., McLellan M. (2015). Pan-cancer network analysis identifies combinations of rare somatic mutations across pathways and protein complexes. Nat. Genet..

[35] Vogelstein B., Papadopoulos N., Velculescu V.E., Zhou S., Diaz L.A., Kinzler K.W. (2013). Cancer Genome Landscapes. Science.

[36] The Cancer Genome Atlas Research Network (2014). Comprehensive molecular profiling of lung adenocarcinoma. Nature.

[37] Vázquez G.H., Holtzman J.N., Lolich M., Ketter T.A., Baldessarini R.J. (2015). Recurrence rates in bipolar disorder: Systematic comparison of long-term prospective, naturalistic studies versus randomized controlled trials. Eur. Neuropsychopharmacol..

[38] Anglemyer A., Horvath H.T., Bero L. (2014). Healthcare outcomes assessed with observational study designs compared with those assessed in randomized trials. Emergencias.

[39] Beaver J.A., Ison G., Pazdur R. (2017). Reevaluating Eligibility Criteria—Balancing Patient Protection and Participation in Oncology Trials. N. Engl. J. Med..

[40] Abernethy A.P., Etheredge L.M., Ganz P.A., Wallace P., German R.R., Neti C., Bach P.B., Murphy S.B. (2010). Rapid-Learning System for Cancer Care. J. Clin. Oncol..

[41] Al-Refaie W.B.M., Vickers S.M.M., Zhong W., Parsons H., Rothenberger D.M., Habermann E.B. (2011). Cancer Trials Versus the Real World in the United States. Ann. Surg..

[42] Meropol N.J. (2016). Overcoming cost barriers to clinical trial participation. Nat. Rev. Clin. Oncol..

[43] Meropol N.J., Buzaglo J.S., Millard J., Damjanov N., Miller S.M., Ridgway C., Ross E.A., Sprandio J.D., Watts P. (2007). Barriers to Clinical Trial Participation as Perceived by Oncologists and Patients. J. Natl. Compr. Cancer Netw..

[44] Yang Y.T., Chen B., Bennett C. (2015). “Right-to-Try” Legislation: Progress or Peril?. J. Clin. Oncol..

[45] Cohen-Kurzrock B.A., Cohen P.R., Kurzrock R. (2016). The right to try is embodied in the right to die. Nat. Rev. Clin. Oncol..

[46] Bray F., Ferlay J., Soerjomataram I., Siegel R.L., Torre L.A., Jemal A. (2018). Global cancer statistics 2018: GLOBOCAN estimates of incidence and mortality worldwide for 36 cancers in 185 countries. CA Cancer J. Clin..

[47] Grossi F., Crinò L., Logroscino A., Canova S., Delmonte A., Melotti B., Proto C., Gelibter A., Cappuzzo F., Turci D. (2018). Use of nivolumab in elderly patients with advanced squamous non–small-cell lung cancer: Results from the Italian cohort of an expanded access programme. Eur. J. Cancer.

[48] Galli G., De Toma A., Pagani F., Randon G., Trevisan B., Prelaj A., Ferrara R., Proto C., Signorelli D., Ganzinelli M. (2019). Efficacy and safety of immunotherapy in elderly patients with non-small cell lung cancer. Lung Cancer.

[49] Corbaux P., Maillet D., Boespflug A., Locatelli-Sanchez M., Perier-Muzet M., Duruisseaux M., Kiakouama-Maleka L., Dalle S., Falandry C., Péron J. (2019). Older and younger patients treated with immune checkpoint inhibitors have similar outcomes in real-life setting. Eur. J. Cancer.

[50] Youn B., Trikalinos N.A., Mor V., Wilson I.B., Dahabreh I.J. (2020). Real-world use and survival outcomes of immune checkpoint inhibitors in older adults with non–small cell lung cancer. Cancer.

[51] Spigel D.R., McCleod M., Jotte R.M., Einhorn L., Horn L., Waterhouse D.M., Creelan B., Babu S., Leighl N.B., Chandler J.C. (2019). Safety, Efficacy, and Patient-Reported Health-Related Quality of Life and Symptom Burden with Nivolumab in Patients with Advanced Non–Small Cell Lung Cancer, Including Patients Aged 70 Years or Older or with Poor Performance Status (CheckMate 153). J. Thorac. Oncol..

[52] Popat S., Ardizzoni A., Ciuleanu T., Dols M.C., Laktionov K., Szilasi M., Califano R., Costa E.C., Griffiths R., Paz-Ares L. (2017). Nivolumab in previously treated patients with metastatic squamous NSCLC: Results of a European single-arm, phase 2 trial (CheckMate 171) including patients aged ≥70 years and with poor performance status. Ann. Oncol..

[53] Passaro A., Spitaleri G., Gyawali B., de Marinis F. (2019). Immunotherapy in Non–Small-Cell Lung Cancer Patients With Performance Status 2: Clinical Decision Making With Scant Evidence. J. Clin. Oncol..

[54] Middleton G., Brock K., Savage J., Mant R., Summers Y., Connibear J., Shah R., Ottensmeier C., Shaw P., Lee S.-M. (2020). Pembrolizumab in patients with non-small-cell lung cancer of performance status 2 (PePS2): A single arm, phase 2 trial. Lancet Respir. Med..

[55] Facchinetti F., Mazzaschi G., Barbieri F., Passiglia F., Mazzoni F., Berardi R., Proto C., Cecere F.L., Pilotto S., Scotti V. (2020). First-line pembrolizumab in advanced non–small cell lung cancer patients with poor performance status. Eur. J. Cancer.

[56] Borghaei H., Paz-Ares L., Horn L., Spigel D.R., Steins M., Ready N.E., Chow L.Q., Vokes E.E., Felip E., Holgado E. (2015). Nivolumab versus Docetaxel in Advanced Nonsquamous Non-Small-Cell Lung Cancer. N. Engl. J. Med..

[57] Brahmer J., Reckamp K.L., Baas P., Crinò L., Eberhardt W.E.E., Poddubskaya E., Antonia S., Pluzanski A., Vokes E.E., Holgado E. (2015). Nivolumab versus Docetaxel in Advanced Squamous-Cell Non–Small-Cell Lung Cancer. N. Engl. J. Med..

[58] Reck M., Rodríguez-Abreu D., Robinson A.G., Hui R., Csőszi T., Fülöp A., Gottfried M., Peled N., Tafreshi A., Cuffe S. (2016). Pembrolizumab versus Chemotherapy for PD-L1–Positive Non–Small-Cell Lung Cancer. N. Engl. J. Med..

[59] Rittmeyer A., Barlesi F., Waterkamp D., Park K., Ciardiello F., von Pawel J., Gadgeel S.M., Hida T., Kowalski D.M., Dols M.C. (2017). Atezolizumab versus docetaxel in patients with previously treated non-small-cell lung cancer (OAK): A phase 3, open-label, multicentre randomised controlled trial. Lancet.

[60] Gandhi L., Rodríguez-Abreu D., Gadgeel S., Esteban E., Felip E., De Angelis F., Domine M., Clingan P., Hochmair M.J., Powell S.F. (2018). Pembrolizumab plus Chemotherapy in Metastatic Non–Small-Cell Lung Cancer. N. Engl. J. Med..

[61] Hendriks L.E., Bootsma G., Mourlanette J., Henon C., Mezquita L., Ferrara R., Audigier-Valette C., Mazieres J., Lefebvre C., Duchemann B. (2019). Survival of patients with non-small cell lung cancer having leptomeningeal metastases treated with immune checkpoint inhibitors. Eur. J. Cancer.

[62] Cortinovis D., Chiari R., Catino A., Grossi F., DE Marinis F., Sperandi F., Piantedosi F., Vitali M., Parra H.J.S., Migliorino M.R. (2019). Italian Cohort of the Nivolumab EAP in Squamous NSCLC: Efficacy and Safety in Patients With CNS Metastases. Anticancer Res..

[63] Grossi F., Genova C., Crinò L., Delmonte A., Turci D., Signorelli D., Passaro A., Parra H.S., Catino A., Landi L. (2019). Real-life results from the overall population and key subgroups within the Italian cohort of nivolumab expanded access program in non-squamous non–small cell lung cancer. Eur. J. Cancer.

[64] Crinò L., Bronte G., Bidoli P., Cravero P., Minenza E., Cortesi E., Garassino M.C., Proto C., Cappuzzo F., Grossi F. (2019). Nivolumab and brain metastases in patients with advanced non-squamous non-small cell lung cancer. Lung Cancer.

[65] Molinier O., Besse B., Barlesi F., Audigier-Valette C., Friard S., Monnet I., Jeannin G., Mazières J., Cadranel J., Hureaux J. (2022). IFCT-1502 CLINIVO: Real-world evidence of long-term survival with nivolumab in a nationwide cohort of patients with advanced non-small-cell lung cancer. ESMO Open.

[66] Franks A.L., Slansky J.E. (2012). Multiple associations between a broad spectrum of autoimmune diseases, chronic inflammatory diseases and cancer. Anticancer Res..

[67] Khan S.A., Pruitt S.L., Xuan L., Gerber D.E. (2016). Prevalence of Autoimmune Disease Among Patients With Lung Cancer. JAMA Oncol..

[68] Cortellini A., Buti S., Santini D., Perrone F., Giusti R., Tiseo M., Bersanelli M., Michiara M., Grassadonia A., Brocco D. (2019). Clinical Outcomes of Patients with Advanced Cancer and Pre-Existing Autoimmune Diseases Treated with Anti-Programmed Death-1 Immunotherapy: A Real-World Transverse Study. Oncologist.

[69] Leonardi G.C., Gainor J.F., Altan M., Kravets S., Dahlberg S.E., Gedmintas L., Azimi R., Rizvi H., Riess J.W., Hellmann M.D. (2018). Safety of Programmed Death–1 Pathway Inhibitors Among Patients with Non–Small-Cell Lung Cancer and Preexisting Autoimmune Disorders. J. Clin. Oncol..

[70] Pasello G., Pavan A., Attili I., Bortolami A., Bonanno L., Menis J., Conte P., Guarneri V. (2020). Real world data in the era of Immune Checkpoint Inhibitors (ICIs): Increasing evidence and future applications in lung cancer. Cancer Treat. Rev..

[71] Shah N.J., Al-Shbool G., Blackburn M., Cook M., Belouali A., Liu S.V., Madhavan S., He A.R., Atkins M.B., Gibney G.T. (2019). Safety and efficacy of immune checkpoint inhibitors (ICIs) in cancer patients with HIV, hepatitis B, or hepatitis C viral infection. J. Immunother. Cancer.

[72] der Welle C.M.C.-V., Peters B.J., Schramel F.M., Klungel O.H., Groen H.J., van de Garde E.M. (2018). Systematic evaluation of the efficacy–effectiveness gap of systemic treatments in metastatic nonsmall cell lung cancer. Eur. Respir. J..

[73] Cramer-Van der Welle C.M., Verschueren M.V., Tonn M., Peters B.J.M., Schramel F.M.N.H., Klungel O.H., Groen H.J.M., van de Garde E.M.W., The Santeon NSCLC Study Group (2021). Real-world outcomes versus clinical trial results of immunotherapy in stage IV non-small cell lung cancer (NSCLC) in the Netherlands. Sci. Rep..

